# Genetic Response to Climatic Change: Insights from Ancient DNA and Phylochronology

**DOI:** 10.1371/journal.pbio.0020290

**Published:** 2004-09-07

**Authors:** Elizabeth A Hadly, Uma Ramakrishnan, Yvonne L Chan, Marcel van Tuinen, Kim O'Keefe, Paula A Spaeth, Chris J Conroy

**Affiliations:** **1**Department of Biological Sciences, Stanford UniversityStanford, CaliforniaUnited States of America; **2**Museum of Vertebrate Zoology, University of CaliforniaBerkeley, Berkeley, CaliforniaUnited States of America

## Abstract

Understanding how climatic change impacts biological diversity is critical to conservation. Yet despite demonstrated effects of climatic perturbation on geographic ranges and population persistence, surprisingly little is known of the genetic response of species. Even less is known over ecologically long time scales pertinent to understanding the interplay between microevolution and environmental change. Here, we present a study of population variation by directly tracking genetic change and population size in two geographically widespread mammal species *(Microtus montanus* and *Thomomys talpoides)* during late-Holocene climatic change. We use ancient DNA to compare two independent estimates of population size (ecological and genetic) and corroborate our results with gene diversity and serial coalescent simulations. Our data and analyses indicate that, with population size decreasing at times of climatic change, some species will exhibit declining gene diversity as expected from simple population genetic models, whereas others will not. While our results could be consistent with selection, independent lines of evidence implicate differences in gene flow, which depends on the life history strategy of species.

## Abstract 2

A novel analysis of paleoclimatic data, fossil abundance and contemporary and ancient DNA from different time periods introduces "phylochronology" and reveals how species respond genetically to climate change

## Introduction

Phylogeography has advanced our understanding of the spatial distribution of genetic diversity within and between species (Avise 2000). However, empirical evidence of temporal change in genetic diversity in a single locality over time has not yet been placed in a population genetic or phylogeographic framework over ecologically long periods of time. In this paper we attempt to determine variation in genetic diversity experienced by populations of two mammalian species in situ and to place that diversity in the context of a changing environment through time. We view this approach as “phylochronology,” or the study of populations in space and time using phylogenetic and population genetic methods. Similar studies have not used such a long temporal record (Pergams et al. 2003), have not considered gene flow (Lambert et al. 2002; Pergams et al. 2003), or have used a spatially averaged sample as a proxy for a single locality (Leonard et al. 2000).

Our study takes advantage of a continuous, well-sampled mammalian fossil sequence spanning the last 3,000 years (Lamar Cave, Yellowstone National Park, Wyoming, United States). Lamar Cave has an extraordinarily complete representation of the local species in the vicinity, with over 10,000 identified mammalian specimens representing over 80% of the mammal species in the local habitat (Hadly 1999). Late-Holocene climatic change, including the Medieval Warm Period (1,150 to 650 years before present [ybp]) and Little Ice Age (650 to 50 ybp) (Soon and Baliunas 2003), affected the local abundances of common small mammals in a manner consistent with their habitat preferences (Hadly 1996).

We focus on two mesic habitat specialists, Microtus montanus (montane vole) and Thomomys talpoides (northern pocket gopher), species that presently are widespread in mountain habitats of western North America. Due to their preferences for wetter habitats, both responded demographically by increasing in relative abundance during wetter climates and declining during warmer climates. M. montanus showed an increase in abundance relative to other common rodents during periods of wet, cool climate in Yellowstone (Hadly 1996). A 40% decline in M. montanus abundance occurred from 2,525 ybp to about 470 ybp during the Medieval Warm Period. Because T. talpoides also demonstrates a preference for mesic montane conditions, shifts in their relative abundance mimic the response seen in *Microtus,* decreasing by 50% between 2,525 and 470 ybp. In addition, T. talpoides showed a significant reduction in body size during this time (Hadly 1997). These data highlight the influence of climatic change on the population dynamics and phenotypic response of these species, especially during warming events.

Although the population responses of T. talpoides and M. montanus are similar, the ways in which they respond to climatic change at the genetic level are predicted to diverge because of differences in dispersal ability and population substructure. Advancement of ancient DNA (aDNA) techniques allows us to investigate directly the impacts of these environmental perturbations on neutral genetic diversity concurrent with these species population responses. We obtained ancient and modern mitochondrial DNA sequences from M. montanus and used previously published data for *T. talpoides.* Although these two species are broadly similar in body size (*M. montanus,* 50–100 g; *T. talpoides,* 75–150 g) and are principally herbivorous, they differ in their natural history. T. talpoides is characterized by low population densities (1–62 gophers/hectare [ha]), a fossorial mode of life, maximum dispersal distances of a few hundred meters, and fiercely territorial behavior (Verts and Carraway 1999). Populations of T. talpoides from Lamar Cave exhibit very little genetic variation through time but considerable genetic differences between present localities (Hadly et al. 1998). This spatiotemporal pattern suggests that late-Holocene gene flow did not influence modern genetic variation of T. talpoides within localities over relatively short time scales (hundreds to thousands of years) despite the absence of obvious migration barriers.

M. montanus achieves higher average population densities (60–186 voles/ha) (Sullivan et al. 2003) than *T. talpoides.* In addition, genetic studies of closely related species and other arvicoline species *(Microtus pennsylvanicus, Microtus longicaudus,* and *Microtus agrestis)* have found little evidence for population subdivision over the scale of hundreds of kilometers (Plante et al. 1989; Conroy and Cook 2000; Bjørnstadt and Grenfell 2001; Jaarola and Searle 2002), as expected from the ability and proclivity of voles to disperse hundreds to thousands of meters, resulting in migration between populations on generational time scales (Jenkins 1948; Lidicker 1985). Such demography, however, implies that historical gene flow may be difficult to detect in M. montanus if only genetic data from the modern animals are used. This is contrary to the pattern expected in T. talpoides because this species has high genetic differentiation between extant populations, and past movement between such populations would be relatively easy to ascertain by the historic presence of unique, divergent haplotypes (Hadly et al. 1998).

The primary advantage of a phylochronologic approach as opposed to a single time slice for understanding mammalian response is the ability to reveal changes in genetic variation through time. This is in contrast to modern genetic studies that seek to reconstruct demographic history based on inferences from past climate or geologic records (e.g., Storz and Beaumont 2002; Lessa et al. 2003). Our study separates demographic and genetic response explicitly, allowing us to understand the microevolutionary forces responsible for the differences in species response over a time scale relevant to evolution within species. This approach is particularly powerful when coupled with environmental data so that perturbations may be linked to organismal response.

In order to reveal these microevolutionary forces from our serial ancient data, it was necessary to explore the influence of sampling from the fossil record and to determine how variation in stochastic evolutionary forces (gene flow, drift, and mutation rate) might influence the record of gene diversity over time. Thus, we combined four methods of estimating population size, determining statistical significance, and assessing gene diversity through time. (1) We derived independent ecological estimates of population size through time from abundances of M. montanus and T. talpoides fossil specimens and modern population densities. (2) We calculated gene diversity over this 3,000-year period to determine the impact of environmental perturbations on genetic effective population size and gene diversity in M. montanus and *T. talpoides.* Unlike previous aDNA work (Consuegra et al. 2002; Hofreiter et al. 2002; Lambert et al. 2002; Orlando et al. 2002; Paxinos et al. 2002), we used mitochondrial DNA sequences for samples taken through time from a single locality. (3) While there have been advances in the use of nuclear markers for ancient genetic analyses, we also confine our analyses to more easily derived mitochondrial DNA (mtDNA) data, thus limiting our analyses to a single locus, usually seen as a neutral marker within mammalian species (Moritz et al. 1987). Our approach also constrains us to the fossil sample sizes from this locality, which are extremely large for ancient DNA studies, but limited relative to population genetic studies. Thus, we assessed the statistical power of a single locus for our empirical data using a neutral population model. (4) We used a neutral population model and serial coalescent simulations to determine whether our observed genetic data reflect our ecological estimates of population size and to evaluate statistical significance in changes of gene diversity through time.

Despite similar population-level responses to climatic change of the late Holocene, we expected differences in gene diversity change for the two species. For *T. talpoides,* we predicted that changes in genetic variation through time would be dominated by drift, as suggested by the modern life history characteristics of small effective population size, low dispersal, and high amounts of population substructure. Therefore, as the ecological effective population size of T. talpoides declined with warmer climates, we expected genetic variation to decline. For *M. montanus,* we predicted that changes in genetic variation through time may be influenced more by migration, as suggested by large effective population sizes, high rates of dispersal, and low amounts of population substructure. As a result, past declines in ecological estimates of population size of M. montanus would not necessarily have resulted in a decrease in genetic variation.

## Results

### Fossil Abundance

**Figure 1:**
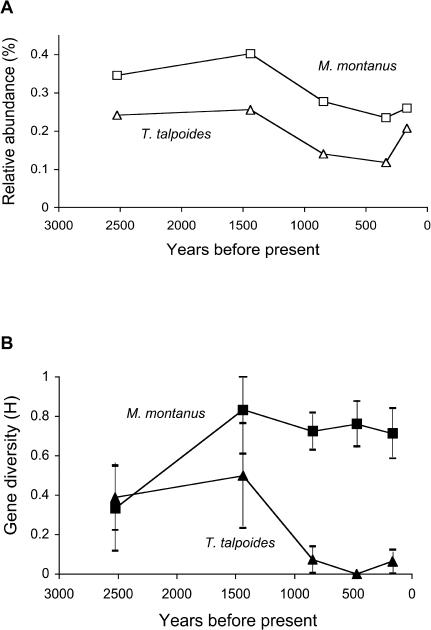
Proportional Population Size and Gene Diversity of M. montanus and T. talpoides (A) Proportional population size (relative abundance) of M. montanus and T. talpoides (*n* = 8,589 fossils) by years before present. (B) Gene diversity (H) of M. montanus and T. talpoides by years before present; 95% confidence intervals are shown. Squares indicate *M. montanus;* triangles indicate *T. talpoides.*

**Figure 2:**
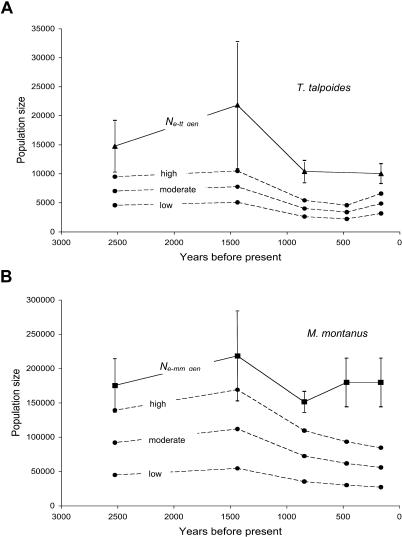
Estimates of *N_e_gen_* and *N_e_ecol_* (A) T. talpoides and (B) M. montanus through time. Circles and dashed lines show *N_e_ecol_* estimates based on low-, high-, and moderate-density estimates. *N_e_gen_* estimates ([A] triangles and [B] rectangles) are based on *θ_S_* estimates from Arlequin. Standard errors for *N_e_gen_* are represented.

Our assessment of population response to climatic change (Figure 1) depends on reconstruction of population size. Fossil relative abundances give a hint of the census size through time while genetic data (gene diversity, Figure 1) should yield independent assessments of the effective population size. The relationship between these measures varies, although most studies suggest that the estimate of effective size derived from ecological data is higher than that derived from genetic data*(N_e_ecol_* >> *N_e_gen_)* (Frankham 1996; Kalinowski and Waples 2002). However, we can convert census size estimates at any point in time into effective size estimates and vice versa. This allows us to compare explicitly ecological and genetic measures of population size. Figure 2 shows *N_e_ecol_* estimates based on low-, high-, and moderate-density estimates for T. talpoides (Figure 2A) and M. montanus (Figure 2B). For M. montanus they range from 218,652 to 436,981 for low-density estimates and from 677,825 to 1,354,650 for high-density estimates. For T. talpoides low-density estimates range from 2,219 to 5,015 and high-density estimates range from 4,586 to 10,488 individuals in the 7-km radius around Lamar Cave.

### Genetic Data: M. montanus

The genetic evidence we have assembled from M. montanus suggests that the sequences we obtained for this study are target mtDNA. Of the 312 bp we sequenced for *M. montanus,* 96.5% of all the mutations were third-position codon changes, with first- and second-position mutations accounting for 3.5% and 0%, respectively. These ratios of variation are concordant with expectations for within-species variation and small overall sequence divergences (Yang and Yoder 1999). Nucleotide base composition is similar to that of other *Microtus* species (Conroy and Cook 2000; Jaarola and Searle 2002), with an excess of adenine (31.2%) and a deficit of guanine (15.7%) (χ^2^; α = 0.71). Most of the mutations are synonymous (97.7%); the transition-to-transversion ratio of the entire data set was 4.1 to 1, which is consistent with expectations for mammalian cytochrome *b* and evolution in other *Microtus* species (Conroy and Cook 2000; Jaarola and Searle 2002). Fossil and modern transition-to-transversion ratios are similar (3.1 and 4.2, respectively). All M. montanus sequences are reciprocally monophyletic (including M. pennsylvanicus as outgroup taxon) and translated successfully. Together with the frequency distribution of our pairwise differences, the prevalence of silent and third-position codon changes, and the standard of obtaining both forward and reverse fragments of overlapping sequence regions, these data permit us to conclude that the genetic diversity we have sampled represents authentic mitochondrial population variation and is unlikely to be from nuclear copies or pseudogenes.

**Figure 3:**
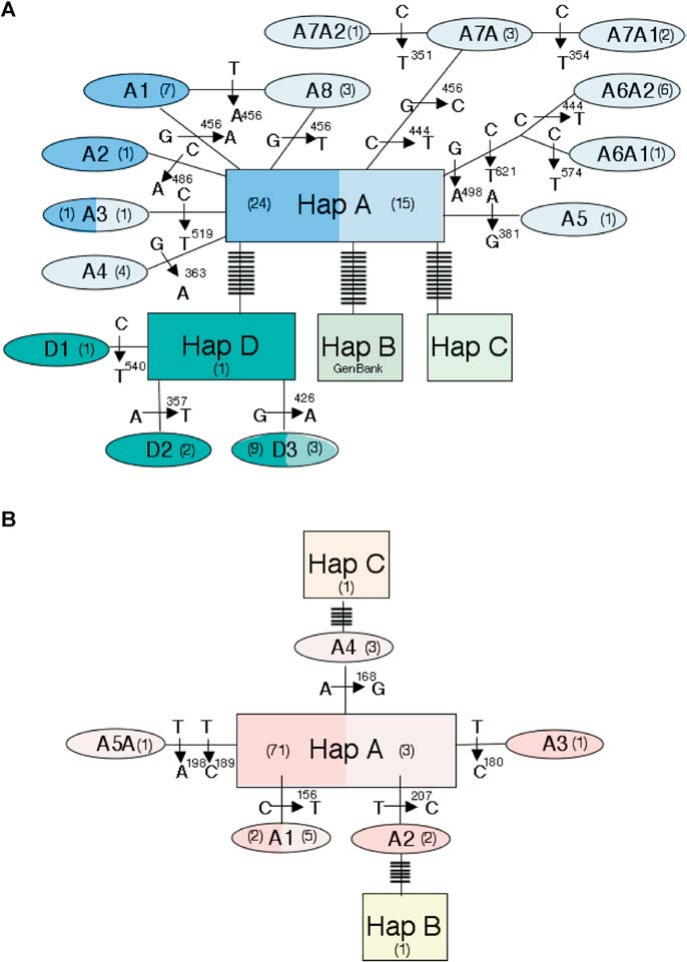
Haplotype Networks for M. montanus and T. talpoides Haplotype networks (Clement et al. 2000) for (A) M. montanus and (B) T. talpoides from Lamar Cave fossils and from modern specimens collected within a 400-km radius of Lamar Cave. Haplogroups for both species are indicated as *A–D.* Each haplogroup within a species is defined by at least 3% sequence divergence within the cytochrome *b* fragment. M. montanus haplogroup *B* is taken from GenBank. Haplogroup *C* is a sample from outside our 400-km radius (NK5897, Mono County, California; Museum of Southwestern Biology #53376). Light shading shows modern samples; dark shading shows fossil samples; bars indicate substitutions; cytochrome *b* sequence positions are indicated by number above base designation. Numbers within parentheses indicate sample sizes for each haplotype.

A total of 282 experiments included 47 fossil extractions and 1,644 PCRs. Eighty-eight percent of our aDNA specimens yielded readable sequence data, with no relationship found between success rate and age of the specimen (*R* ^2^ = 0.004, not significant). All but one (out of 121) of the extraction controls were negative. When sequenced, this extraction blank BLASTed similar to *Montanus townsendii,* a taxon we had never worked on in the facility; this sequence has not since been amplified in the lab, and that extraction was not used further. Out of 87 successfully amplified samples and one sequence obtained from GenBank (AF119280), we identified 17 haplotypes within four haplogroups *(A–D)* of M. montanus (Figure 3A). The distribution of haplotypes within haplogroups suggests that our groups are defined appropriately. Each haplogroup was defined by at least 3% sequence divergence (≥10 bp) from other haplogroups in the 312-bp cytochrome *b* fragment. The majority of individuals (98.8%) fall within haplogroups *A* and *D,* with 84% of the samples within one substitution of the locally ancestral haplotype *A* (Figure 3A).

The maximum uncorrected sequence divergence for our complete spatial and temporal data set was 4.5%, demonstrated between haplogroups *A* and *B.* Given that the highest average rodent divergence rate for cytochrome *b* is 6% to 10% per million years (Irwin et al. 1991), these haplogroups have been evolving separately for at least 450,000 years. A similar age (422,000 years) is found when using a rate of 2.3% per million years for third-position transversions (Conroy and Cook 1999). The maximum uncorrected sequence divergence of M. montanus from throughout the Lamar Cave temporal sequence was 4.2%. The maximum sequence divergence of 19 modern individuals from populations of this species within Yellowstone National Park and surroundings was 3.8%.

### Genetic Data: T. talpoides

Protocols for T. talpoides are found in Hadly et al. (1998). A haplotype network of this species shows three haplogroups and a total of eight haplotypes from 76 specimens (Figure 3B). For *T. talpoides,* 98.0% of all the mutations were third-position codon changes, with first- and second-position mutations accounting for 0% and 2.0%, respectively. Nucleotide base composition shows an excess of thymine (34.7%) and a deficit of guanine (11.4%). All mutations were synonymous; the transition-to-transversion ratio of the entire data set was 4.0 to 1.

### Gene Diversity through Time

Estimates of gene diversity, nucleotide diversity, and number of segregating sites differed between M. montanus and T. talpoides (Table 1). The estimates for M. montanus were higher than those for *T. talpoides,* as predicted by life history traits including higher ecological effective population size and higher dispersal between populations.

**Table 1:**
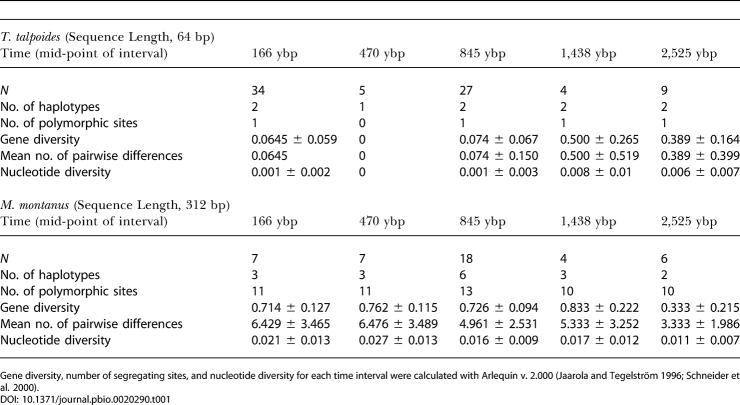
Summary Statistics, Sample Sizes, Sequence Length, and Number of Haplotypes for the Ancient DNA Samples for T. talpoides and M. montanus from Lamar Cave, Wyoming

Gene diversity, number of segregating sites, and nucleotide diversity for each time interval were calculated with Arlequin v. 2.000 (Jaarola and Tegelström 1996; Schneider et al. 2000)

Our raw data on these species show similar relative abundance patterns but disparate trends in gene diversity (see Figure 1). These patterns cannot be linked directly to relative abundance because gene diversity estimates depend on true population size as well as sampling. We investigate how both of these parameters impact the observed trend in the following sections.

### Comparing Ecological and Genetic Estimates of Effective Size

Ecological estimates of population size for *T. talpoides (N_e-tt_ecol_)* exhibit the same trend as the genetic estimates *(N_e-tt_gen_),* namely a population size decline of >50% after 1,500 ybp (see Figure 2A). For *T. talpoides, N_e-tt_gen_* is consistently higher than *N_e-tt_ecol_.* When compared to those for M. montanus (see Figure 2B), the estimates of total ecological and genetic effective population size are much lower. Additionally, unlike with *M. montanus,* the ecological and genetic effective sizes follow similar trends through the entire time period sampled, indicating that T. talpoides is acting as a closed population.

For *M. montanus,* the estimates of effective size derived from the ecological data *(N_e-mm_ecol_)* are lower than those derived from genetic data *(N_e-mm_gen_)* for all time points (see Figure 2B). While the estimates are not expected to be identical, comparison of their trends is instructive. Both genetic and ecological estimates follow similar trends between 2,525 and 845 ybp, after which the two estimates follow opposite trajectories. While the ecological size decreases by 50%, the genetic estimates show an initial decline of 30%, followed by an increase in population size equivalent to the pre–1,438-ybp level. This demonstrates that although the population is not recovering ecologically, it does recover genetically from population decline between 1,438 and 845 ybp.

### Effects of Sampling

**Figure 4:**
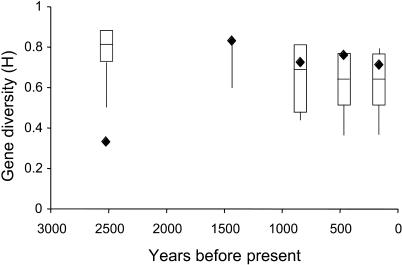
Expected and Observed Gene Diversity of M. montanus Boxes represent the 95th, 50th, and fifth percentiles for expected gene diversity of M. montanus given estimates of *N_e-mm_ecol_* at 2,525, 1,438, 845, 470, and 166 ybp and the associated sample sizes (*n* = 7, 7, 18, 4, and 6) based on the Ewens sampling distribution (assumed mutation rate = 4% per million years per bp for a 312-bp fragment). Bars represent the 95th and fifth percentiles for a sample size of 100 at the same points in time. Diamonds represent observed gene diversity from empirical genetic data. The empirical data for each time unit fall within the expected ranges of gene diversity, except those for 2,525 ybp, which are much too low for the seven samples to detect, suggesting that observed gene diversities are not limited by sample size.

For *M. montanus,* our observed data were within the 95% confidence intervals for both sets of simulations (*n_sample_* and *n* = 100; mutation rate = 4% per million years; moderate density values used to calculate abundance) for all time points except for 2,525 ybp, where observed gene diversity was significantly lower than could have been calculated given our sample size (Figure 4). Since the observed gene diversity is lower than expected, we repeated simulations for five additional combinations of mutation rate and abundance (low mutation rate, high abundance; low mutation rate, moderate abundance; low mutation rate, low abundance; moderate mutation rate, low abundance; high mutation rate, low abundance). Results revealed that the observed gene diversity at 2,525 ybp was within the lower fifth percentile of the predicted distribution for two of the five combinations (when both mutation rate and abundance were low and for low mutation rate, moderate abundance). The overlap between the 95% confidence intervals for both sets of simulations suggests that sampling bias does not significantly impact the observed patterns of gene diversity except at 1,438 ybp (*n* = 4), suggesting that we do not have sufficient power to detect processes at this time period.

Although the gene diversity for T. talpoides is not different given expectations from a closed population, we attempted to determine the statistical limitations of these data. Investigation of the effects of sampling for T. talpoides revealed that given the smaller number of base pairs (64 bp), we do not have enough statistical power to reject the null hypothesis. For every sampling time point, the Ewens distribution predicted that only one haplotype would be present in the genetic samples (unpublished data). As a result, the predicted gene diversity was zero for all time points. Because the observed gene diversity for T. talpoides was higher than predicted we also simulated five combinations of mutation rate and abundance, which could result in a higher predicted diversity (high mutation rate, high abundance; high mutation rate, moderate abundance; high mutation rate, low abundance; moderate mutation rate, high abundance; low mutation rate, high abundance) for 166 ybp (*n* = 34). Results for all five combinations predicted presence of a single haplotype. Since we do not have adequate statistical power given the genetic data for *T. talpoides,* we did not conduct significance tests for this species. However, the observed values of gene diversity are not unexpected from dynamics within a closed population.

### Significance of Changes in Gene Diversity in M. montanus

**Figure 5:**
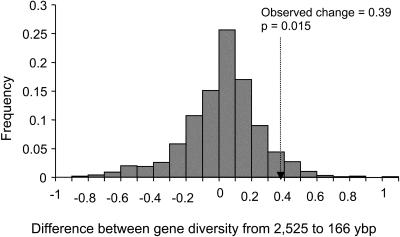
Distribution of Change in Gene Diversity for M. montanus between 2,525 and 166 ybp, Based on Serial Coalescent Simulations Sampling is modeled at two points in time. *N_e-mm_ecol_* estimates from Figure 2 are used to specify demographic history. Eight of the nine combinations of mutation rate and density allow us to reject the null hypothesis for a closed population (Table 2). This figure illustrates simulation results for moderate density and moderate mutation rate (4% per million years per bp). The probability of the observed change (shown by dashed arrow) is significant (*p* = 0.015).

For eight of the nine combinations of mutation rate and effective size, we could reject the null hypothesis (closed population, no selection, changes in abundance inferred through fossil abundance) based on the observed change in M. montanus gene diversity throughout the entire time series (2,525 to 166 ybp) (Table 2). The expected distribution of change in gene diversity given the null hypothesis and based on moderate M. montanus densities and moderate mutation rate is shown in Figure 5 (Table 2 shows all combinations), along with the observed change. However, given the observed change in gene diversity specifically between 2,525 and 845 ybp, we were able to reject the null hypothesis for all nine combinations of mutation rate and effective size. These results suggest that M. montanus was not acting as a closed population during this period of time. Because the serial coalescent model presented here does not discriminate between selection and migration, either of these processes could have caused the observed change in gene diversity.

**Table 2:**
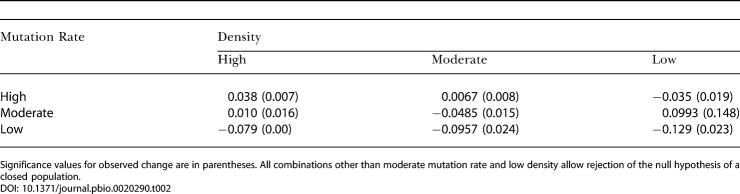
The Average (over 1,000 Simulations) Expected Change in Gene Diversity for Nine Combinations of Density and Mutation Rate for M. montanus between 2,525 and 166 ybp

Significance values for observed change are in parentheses. All combinations other than moderate mutation rate and low density allow rejection of the null hypothesis of a closed population

Our results may depend on our assumptions of equilibrium population size prior to 2,525 ybp. We investigated sensitivity to this assumption by modeling a population bottleneck in M. montanus prior to 2,525 ybp. We modeled population reduction to 104,577 (0.75 × *N_e-mm_ecol2525ybp_*), 69,718 (0.5 × *N_e-mm_ecol2525ybp_*), and 34,859 (0.25 × *N_e-mm_ecol2525ybp_*) prior to 2,525 ybp. For a 75% bottleneck prior to 2,525 ybp, we were no longer able to reject the null hypothesis of a closed population. These results indicate that an extreme bottleneck where population size was reduced to 75% or more might result in the observed change in gene diversity. Our simulations reveal that unless an extreme bottleneck happened prior to 2,525 ybp, we can be confident that the observed change in gene diversity is not likely to be from events that occurred immediately prior to our historic data, and thus is due to migration or selection.

Coalescent simulations used to investigate the significance of the observed gene diversity value for the modern samples demonstrated that the null hypothesis of past population size change could be rejected at only two of the nine mutation rate and density combinations. These results reveal that given data from only the modern samples, it was not possible to reject the null hypothesis of past population size change in *M. montanus.* Historic genetic data allow us to discriminate between population processes over millennia much better than do modern data alone.

### Other Evidence for Migration

Independent lines of genetic and demographic evidence also point to the influence of gene flow in M. montanus populations. Estimates of *β*-diversity (used here to measure haplotypic turnover) from haplotypic data reveal that turnover was highest between 2,525 and 845 ybp (*β_2525–845ybp_* = 3; *β_845–166ybp_* = 1.5; and *β_2525–166ybp_* = 1.5). Closer examination of the haplotypic distributions demonstrates that five novel haplotypes appeared by 845 ybp, three of which are ≥3.2% different from the most common haplogroup *(A)* (Figure 3A) at 2,525 ybp, further implicating gene flow between 2,525 and 845 ybp.

## Discussion

Our results demonstrate different genetic responses by two species of small mammals to changes in population size driven by climatic change. Fossil abundance data reveal population decline for both T. talpoides and M. montanus between 1,438 and 470 ybp, a period spanning the Medieval Warm Period (Hadly 1996). For *T. talpoides,* the genetic response is directly related to changes in population size: Decrease in population size results in lowered gene diversity. M. montanus demonstrates the opposite relationship: A decrease in population size (between 1,438 and 166 ybp) results in an increase in gene diversity. We attempted to statistically validate our results by the use of serial coalescent simulations to demonstrate that the change in gene diversity of M. montanus between 2,525 and 845 ybp is significantly different from that expected based on the decrease in ecological estimates of population size. Taken together, these results indicate a departure from conditions of equilibrium (closed population without selection) for *M. montanus.* Our results have the following possible explanations: (1) the sampling area for fossils changed, (2) the local population size expanded, (3) selection occurred, and/or (4) gene flow occurred.

### Selection versus Gene Flow

Results from all three of our analyses suggest that gene flow could be responsible for the patterns in gene diversity observed in our empirical data. Additionally, recent results of experimental studies of density dispersal dynamics in the root vole, *Microtus oeconomus,* indicate that migration occurs most frequently in and between low-density patches (Andreassen and Ims 2001). These results indicate that density and dispersal in voles may be inversely related, a finding that is consistent with our results.

An alternative explanation is that selection is governing the observed gene diversity patterns. While cytochrome *b* may not be under intense selection (Irwin et al. 1991), it is linked to other portions of the mitochondrial genome that may be selectively advantageous in particular environments. Using cytochrome *b* as a marker for the accumulations of adaptations elsewhere on the genome may yield information about the effects of selection on local populations through time. Further exploration is necessary to investigate and identify the presence of locally adapted mtDNA and the rates of evolutionary change necessary to produce the variation in gene diversity we have observed (e.g., Pergams et al. 2003).

### Conclusions

Here we demonstrate, using a phylochronologic approach, that it is possible to distinguish the dynamic processes that govern gene diversity over relatively short time scales (hundreds to thousands of years). We have documented environmental change, population response, genetic diversity change, and the correlations between the three. Without serial data, we would capture just a single record of these historic processes: modern genetic diversity. Although it is possible to hypothesize about historic events using modern data, phylochronology affords a unique look into the past and the potential ability to separate cause from effect. In particular, we show that M. montanus has a history recording responses both within populations (fluctuations in population size, possible selection) and between populations (gene flow). Discrepancy between the ecological and genetic estimates of population size and significant changes in haplotypic diversity prior to the Medieval Warm Period implicate increased gene flow into the Lamar Cave M. montanus population. Additionally, the observed haplotypic turnover in the Yellowstone population during this period suggests that as abundance of M. montanus declined through the last 845 years, relatively more individuals carried newly introduced haplotypes.

Our results indicate that the presently observed widespread genetic variation across the geographic range in this species arose not because gene flow was equivalent through all populations through time, but because during particular time periods, certain local populations (and/or genotypes) declined while others expanded. Our data show that even with a prolonged ecological population size decline, the genetic diversity of M. montanus was maintained. In contrast, gene flow has not played a significant role in the recent genetic history of *T. talpoides.* This species, instead, responded more as a closed population over this time. The disparate nature of population response to climatic change of these two species is likely due to differences in demographic dispersal patterns between their populations. Such differences in species demography have resulted in differential genetic response to climatic change, even when ecological response is similar. Thus, genetic response to environmental change can be viewed as “individualistic,” similar to unique adjustments of species ranges (Root et al. 2003). Life history traits such as dispersal ability contribute to the overall gene diversity of species in both space and time. If life history has such a large impact for common species, such differences will be particularly important in understanding how entire communities are affected by global change. Ultimately, knowledge from such analyses will lead to distinct, and perhaps predictable, patterns of species persistence through climatic changes, insights that will prove invaluable to future conservation of biodiversity.

## Materials and Methods

### 

#### Fossil locality

Lamar Cave contains well-stratified, thoroughly radiocarbon-dated deposits, which display high fidelity to the local mammalian community (Hadly 1996, 1999). The most common animals from Lamar Cave are also the most common in the sagebrush grassland ecosystem in which Lamar Cave is located. Relative abundances are based on the entire data set of 10,597 specimens (except for those in Figure 1A, which uses the five most common small mammals [*n* = 8,589]) and are concordant with expectations of taxonomic diversity in montane mammal communities of western North America (Hadly and Maurer 2001). The cumulative number of bones in Lamar Cave is correlated with time, demonstrating a constant “rain” of bones from the past to the present environment (*R* ^2^ = 0.86; unpublished data).

#### Age assignment

The historical *Microtus* samples encompass 15 of 16 radiocarbon-dated stratigraphic levels from Lamar Cave (Hadly 1996), with a maximum radiocarbon age of 2,860 ± 70 ybp (CAMS-20356). Data from the stratigraphic levels were pooled into five discrete intervals for Lamar Cave representing the last 3,000 years (Hadly 1996). Interval boundaries were based on the stratigraphic pattern of deposition observed during excavation as well as a detailed radiocarbon chronology. Thus each interval represents a biologically significant packet of specimens. Age for each interval was assigned as the midpoint of the span of the calibrated radiocarbon ages (Hadly 1996).

#### Ecological estimates of population size

Absolute population sizes in both ancient and modern communities are difficult to estimate. However, relative abundance changes of the small mammals are consistent with the climatically caused changes in habitats and the habitat preferences documented by modern trapping data proximate to the fossil site (Hadly 1999, Figure 1A). By calculating the area preferred by M. montanus and T. talpoides using the geographical information system, we were able to standardize the relationship between taphonomy and population size in these species. This was possible because the collection radius of the fossils from Lamar Cave has been documented to be less than 7 km by using strontium isotopes (Porder et al. 2003). We then estimated ecological effective population size from relative abundance through time, current population density, and the current area of preferred habitat in the collection radius. The current area of M. montanus habitat within the 7-km radius totals 1,992 ha; for T. talpoides it totals 971 ha. High, moderate, and low densities for T. talpoides (Verts and Carraway 1999) and M. montanus (Sullivan et al. 2003) were used in association with the corresponding areas of occupied habitat to estimate current census size. Because the rate of accumulation of bones through time is constant, the current census size was indexed against the percentage of *Microtus* bones from the uppermost level of Lamar Cave and used to calculate historic census sizes through time.

In order to compare ecological estimates of effective population size to genetic estimates, we estimated mitochondrial effective population size assuming that *N_e_/N_census_* = 0.5 (Storz et al. 2002; estimates from small mammals), and given that mitochondrial effective size is *N_e_*/4. Our estimates of *N_e_* were based entirely on relative fossil abundance and current ecological data and assume a mitochondrial effective size; thus we labelled them *N_e-tt_ecol_ (T. talpoides)* and *N_e-mm_ecol_ (M. montanus).*

#### Genetic data from *M. montanus*

From the fossil material, we used the upper first molar (from only one side of the jaw per level) to avoid sampling the same individual multiple times. Some *Microtus* species are cryptic with respect to these teeth. Genetic diagnosis indicated that these 78 fossil samples are derived from multiple arvicoline species, with a total of 47 M. montanus specimens (see Table 1). Fossil samples (*Microtus* molariform teeth) ranged from 3.5 to 15.3 mg (average, 8.8 mg). We used two previously described extraction methods on the fossil teeth (Hadly et al. 2003). We obtained a 312-bp fragment of mitochondrial cytochrome *b* from ancient samples (*n* = 47) using the following primers: forward primers (5′–3′), CLETH 37 TAY AAY ATA ATY GAA ACH TGA A (5′ end of cyt *b* 319 equals Mus musculus 14458), CLETH 37L AYG GMT CTT AYA ACA TAA TCG AAA CAT G (cyt *b* 311, M. musculus 14450), MMONT 1 CAG TAA TTA CAA AYC TWC TAT CA (cyt *b* 452, M. musculus 14591), and MMONT 3 AGT GAA TCT GAG GGG GCT TCT CAG TAG A (cyt *b* 485, M. musculus 14621); reverse primers (5′–3′), ARVIC 08 CAG ATY CAY TCY ACT AGT GTT G (cyt *b* 473, M. musculus 14612), ARVIC 08L CTC AGA TTC ACT CTA CTA GTG TTG TG (cyt *b* 471, M. musculus 14610), MMONT 4 TTR TTT GAT CCT GTT TCG TGT AGG AAT A (cyt *b* 595, M. musculus 14631), and MMONT 2L TTG ACT GTG TAG TAA GGG TGA AAT GGG A (cyt *b* 653, M. musculus 14792). Attempts were made to amplify the region in two overlapping fragments using CLETH37/ARVIC 08, CLETH37L/ARVIC 08L, and MMONT 1/MMONT 2L. However, the low rate of success for MMONT 1/MMONT 2L (40%) in the fossil samples necessitated the breaking of the second fragment into two overlapping fragments using MMONT 1/MMONT 4 and MMONT 3/MMONT 2L.

Ancient DNA samples were run on an ABI PRISM 310 Genetic Analyzer in the post-PCR lab, and modern DNA samples were run on an ABI PRISM 377 Sequencer in a separate sequencing facility (Protein and Nucleotide Facility, Beckman Center, Palo Alto, California, United States). Fragments were sequenced in both directions, primer regions were overlapped, and sequences with any ambiguous sites were rerun until completely resolved in order to provide additional corroboration and eliminate ambiguity.

We adhered to strict extraction and amplification protocols (Hadly et al. 2003). The protocol further included (1) independent sequence corroboration of two samples (J. Mountain lab, Anthropological Sciences, Stanford University), (2) processing of modern samples using personnel and reagents in another lab (D. Petrov lab, Biological Sciences, Stanford University) all physically separate from the aDNA facility, (3) monitoring contamination with several extraction and PCR controls, (4) primer design specific to arvicoline species, and (5) no prior or concurrent history of working with murid species in any of the DNA facilities involved.

Modern (spatial) genetic sampling was obtained from a variety of sources including modern skins, modern liver tissue, museum skins, and teeth derived from modern raptor pellets. DNA was successfully extracted from 16 modern skins *(n* = 13 *M. montanus; n* = 3 other *Microtus)* and 12 teeth *(n* = 5 *M. montanus; n* = 7 other *Microtus)* from the vicinity (within 10 km) of the fossil site. In addition, DNA was successfully extracted from 51 modern specimens (liver tissue and museum skins) collected within a 400-km radius of Lamar Cave. Of these 51 samples, 22 were derived from museum skins *(n* = 21 *M. montanus; n =* 1 *M. longicaudus)* and 29 from liver tissue *(n* = 9 *M. montanus; n* = 18 other *Microtus)* of specimens trapped in the field. We followed the animal tissue protocol using the Qiagen Dneasy Tissue Kit (Qiagen, Valencia, California, United States) on 6.5 mg of liver samples and 7.5 mg of tooth samples (*n* = 3). For museum samples, DNA was extracted from the ventral skin incision (0.5 to 10.3 mg; average, 2.7 mg). We amplified the entire cytochrome *b* gene (1,143 bp) for some modern skins (*n* = 13) and liver tissue (*n* = 29) with the following primers: forward primers (5′–3′), MVZ 05 CGA AGC TTG ATA TGA AAA ACC ATC GTT (cyt *b* −51, M. musculus 14088) and ARVIC 07 AAA GCC ACC CTC ACA CGA TT (cyt *b* 514, M. musculus 14653); reverse primers (5′–3′) MICRO 06 GGA TTA TTT GAT CCT GTT TCG T (cyt *b* 602, M. musculus 14741) and VOLE 14 TTT CAT TAC TGG TTT ACA AGA C (cyt *b* 1170, M. musculus 15309).

DNA was extracted from a total of 81 modern *Microtus* samples, and 79 of those yielded successful amplification. Of these 79 samples, 48 were positively identified as M. montanus. Of the M. montanus samples, 41 specimens were successfully haplotyped. Sequences have been deposited in GenBank (see Supporting Information).

#### Genetic data from *T. talpoides*

We have built upon the previously published T. talpoides data set (Hadly et al. 1998) with additional temporal sampling (*n* = 3) (see Table 1). T. talpoides from Lamar Cave demonstrated remarkable continuity in gene diversity through time, with only three haplotypes present, all of which differ from each other by one synonymous third-position mutation. The majority of the fossil T. talpoides specimens are from haplotype *A* (83%), which is not found elsewhere in a 400-km radius around Lamar Cave. This constancy in the genetic lineage of T. talpoides within a single locality persists in spite of climatic changes and concurrent significant population and body size changes (Hadly et al. 1998).

#### Data analysis

Arlequin v. 2.000 (Jaarola and Tegelström 1996; Schneider et al. 2000) was used to calculate gene diversity, number of segregating sites, and nucleotide diversity for each time interval (see Figure 1B and Table 1). Additionally, genetic data were used to estimate the mitochondrial effective population size *(N_e_)* for all points in the past. Assuming a neutral model of molecular evolution, *θ_S_* (where *S* is the number of segregating sites; *θ_S_* = 2 *N_e_μ*, where *μ* is the mutation rate for the complete sequence per generation) was used to estimate *N_e_. θ_S_* was preferred over other estimators of *θ* since *θ_H_* is biased for single locus estimates, *θ_k_* does not incorporate sequence information, and *θ_π_* has higher variance. These estimates are determined entirely from genetic data; thus we label them *N_e-tt_gen_ (T. talpoides)* and *N_e-mm_gen_ (M. montanus).* We assumed a range of *μ* (2% [low], 4% [moderate], or 10% [high] per million years per bp) (Table 2). Additionally, to estimate haplotype turnover we calculated *β*-diversity (used traditionally in ecology to measure species turnover) using Cody's index (Cody 1968). For *β*-diversity each haplotype was treated as a “species.”

#### Effects of sampling

Given the general limitations of obtaining aDNA sequence data, sample sizes will always present challenges to ancient population genetic studies. To investigate whether our samples are adequate for addressing temporal gene diversity in both species, we used a neutral population model to evaluate expected diversity. Ewens (1972) derived expressions for the sampling distribution characterizing a closed population of size *N,* a gene with mutation rate *μ,* and an infinite alleles model given a sample size *n.* Here we use the Ewens distribution to ascertain the ability of our data to detect variation in values of gene diversity. In addition, this distribution allows us to (1) predict the distribution of expected gene diversity at each point in time (independently) given estimates of *N_e_ecol_,* a moderate mutation rate of 4% per million years, typed sequence length (312 bp for M. montanus and 63 bp for T. talpoides), and sample size (*M. montanus: n_166ybp_* = 7, *n_470ybp_* = 7, *n_845ybp_* = 18, *n_1438ybp_* = 4, and *n_2525ybp_* = 6; *T. talpoides: n_166ybp_* = 34, *n_470ybp_* = 5, *n_845ybp_* = 29, *n_1438ybp_* = 4, and *n_2525ybp_* = 11) and (2) predict change in the expected gene diversity distribution for a large sample size of 100. A modified version of MONTE CARLO (Slatkin 1994, 1996) was used to generate possible allele configurations for a given set of parameters *(θ, K,* and *n),* and gene diversity was calculated for each configuration. Simulations were repeated 1,000 times for each sampling time point, resulting in the 95% confidence intervals for the distribution of predicted gene diversities given a closed, selectively neutral population through time.

#### Serial coalescent simulations

We assessed the significance of the observed changes in gene diversity between time points using simulations. The serial coalescent was used as a framework to model M. montanus evolution during the past 2,525 years. Simulations were repeated 1,000 times to generate genetic data and changes in gene diversity for the null hypothesis. The significance of the observed changes was then inferred by comparing it to the generated null values (we thus used a Monte Carlo significance test). Estimates of *N_e_ecol_* were used to set up a null hypothesis specifying population size change through time in a closed population. We assumed that past changes in population size between intervals were due to exponential growth or decline. The effective population size at two time points and the time between the points was used to calculate a growth rate. The estimated growth rates were *r_1438–2525ybp_* = −0.000178, *r_845–1438ybp_* = 0.000732, *r_470–845ybp_* = 0.0004385, and *r_166–470ybp_* = 0.0003212. The null hypothesis thus corresponds to effective population sizes at particular points in the past and exponential growth or decline between those intervals, and represents an ecologically realistic description of the past 2,525 years. We assumed a constant population size prior to 2,525 ybp, as we have no data before this point in time. The coalescent program SIMCOAL (Excoffier et al. 2000) was modified to incorporate temporal sampling (also known as heterochronous sampling): *n_1_* samples modeled back in time, with *n_2_* samples added to the genealogy at a time point *t_1_* generations in the past. The serial coalescent (Rodrigo and Felsenstein 1999; Drummond et al. 2003) has been used to estimate parameters such as *μ* for HIV and ancient mtDNA (most recently using an MCMC approach; Drummond et al. 2002; Lambert et al. 2002). In this paper, we present what we believe to be its first application as a simulation tool used to predict change in gene diversity for the null model of population size change described above. Running the model 1,000 times provides the expected distribution for change in gene diversity. Using *N_e-mm_ecol_* estimates based on high-, moderate-, and low-density estimates for M. montanus (186, 126, and 60 voles/ha; Sullivan et al. 2003) and a high, moderate, and low mutation rate (*μ* = 10%, 4%, and 2% per million years per bp; sequence length = 312 bp; finite sites mutation model, no rate heterogeneity), we investigated the significance of observed changes in gene diversity at all nine parameter combinations over the entire time span (2,525 to 166 ybp) and between 2,525 and 845 ybp (spanning the Medieval Warm Period).

Additionally, we also investigated the relevance of temporal data to our ability to reject the null hypothesis. Significance tests were repeated assuming the observed data were from only the most recent genetic samples. Again, simulations were repeated for all nine combinations of mutation rate and density estimates.

## Supporting Information

### Accession Numbers

Sequences for the successfully haplotyped M. montanus specimens described in Materials and Methods have been deposited in GenBank under accession numbers AY660606 to AY660629.

## Abbreviations

aDNA: ancient DNA
bp: base pair
ha: hectare
mtDNA: mitochondrial DNA
*N_e_ecol_*: estimate of effective size derived from ecological data
*N_e_gen_*: estimate of effective size derived from genetic data
*N_e-mm_ecol_*: *N_e_ecol_* for *M. montanus*
*N_e-mm_gen_*: *N_e_gen_* for *M. montanus*
*N_e-tt_ecol_*: *N_e_ecol_* for *T. talpoides*
*N_e-tt_gen_*: *N_e_gen_* for *T. talpoides*
ybp: years before present
